# Vigorous Physical Activity Among Tweens, VERB Summer Scorecard Program, Lexington, Kentucky, 2004-2007

**Published:** 2011-08-15

**Authors:** Moya L. Alfonso, Robert J. McDermott, Zachary Thompson, Carol A. Bryant, Anita H. Courtney, Jenna L. Davis, Yiliang Zhu, Jeffery A. Jones

**Affiliations:** Georgia Southern University, Statesboro, Georgia, and University of South Florida College of Public Health, Tampa, Florida; Florida Prevention Research Center, University of South Florida College of Public Health; University of South Florida College of Public Health, Tampa, Florida; University of South Florida College of Public Health, Tampa, Florida; University of South Florida College of Public Health, Tampa, Florida; University of South Florida College of Public Health, Tampa, Florida; University of South Florida College of Public Health, Tampa, Florida; University of Kentucky College of Public Health, Lexington, Kentucky

## Abstract

**Introduction:**

Empirical examinations of the efficacy of community-based programs to increase and sustain physical activity among youth are lacking. This study describes changes in vigorous physical activity during a 3-year period among children aged 9 to 13 years (tweens) in Lexington, Kentucky, following introduction of the VERB Summer Scorecard (VSS) intervention.

**Methods:**

A community coalition, guided by a marketing plan that addressed motivators for tweens to participate in physical activity, designed and implemented VSS. Youth used a scorecard to monitor their physical activity, which was verified by adults. There were 3,428 students surveyed in 2004; 1,976 in 2006; and 2,051 in 2007 (mean age for 2004, 2006, and 2007, 12 y). For each year, we performed *Χ* ^2^ tests and computed summary statistics for age, sex, and grade. Chi-square tests and cumulative logit models were used to analyze physical activity trends among VSS participants, VSS nonparticipants, and a reference group.

**Results:**

The proportion of youth who reported frequent vigorous physical activity increased from 32% in 2004 to 42% in 2007. The proportion of VSS participants with moderate or high levels of vigorous physical activity increased by approximately 17 percentage points, more than twice the proportion of nonparticipants.

**Conclusion:**

Interventions such as VSS may empower communities to take action to encourage greater physical activity among youth.

## Introduction

The prevalence of overweight and obesity among US youth increased 23% during the past 3 decades (1). Approximately 20% of children aged 6 to 11 years and 18% of youth aged 12 to 19 years are obese (2). The risk of cardiovascular disease and incidence of chronic illnesses such as diabetes have increased during childhood as a result of increasing rates of overweight and obesity in children (3-5). Some experts estimate that 42% to 63% of overweight youth will become overweight adults (6), and overweight and obese adults have increased risk of multiple health complications (7). The pervasiveness and long-term ramifications of overweight and obesity make them serious public health concerns.

One way to reduce the risk of overweight is by being physically active (8). Patterns of physical activity (PA) established in youth persist into adulthood (9-12). Therefore, active youth tend to remain active during their teenage years (13,14). However, there is a risk of declining PA between childhood and adolescence, which is more pronounced among girls than boys (14-17). Interventions that encourage PA are crucial during the preteen years (9).

The Centers for Disease Control and Prevention (CDC) introduced the VERB — It's What You Do campaign, which ran from 2002 to 2006 (18,19). The campaign promoted activities that "tweens" (ie, children aged 9-13 y) could do with few time or place restrictions. Rather than focusing directly on exercise, VERB emphasized activities that the literature indicates motivate youth: spending time with friends, trying new activities, and having fun (20-22). VERB relied on paid advertising, partnership efforts, and other marketing strategies for promoting the program to tweens (18,19). Additional audiences were parents and other influential adults, including teachers, youth leaders, physical and health education professionals, pediatricians, health care providers, and coaches (18).

Missing from the national VERB campaign were local implementation strategies, particularly outside of schools. This exclusion is problematic during summer, when body mass index may increase (23,24) because of more time available for watching television, playing computer-based games, and participating in other sedentary activities, as well as the influence of decreased structure (24). Therefore, summer is an appropriate time to intervene in an effort to change tweens' participation in PA.

The VERB Summer Scorecard (VSS) intervention was created in Lexington, Kentucky, in 2004 by the Lexington Tweens Nutrition and Fitness Coalition, pilot-tested, and implemented as a local extension of the national VERB campaign. VSS enabled youth to try new and fun activities during summer (25,26) and was designed to increase opportunities for vigorous PA (VPA). Empirical examinations of the efficacy of community-based programs to increase and sustain PA among youth are lacking (9,11). The purpose of this study was to describe changes over time (2004-2007) in VPA among tweens on the basis of their participation status in VSS.

## Methods

### Setting

Lexington is Kentucky's second-largest city. The 2000 census indicated that Lexington had 260,512 people (27), 108,288 households (28), and 62,915 families (28). Its racial composition was 81.0% white, 13.5% African American, 0.2% Native American, 2.5% Asian, and 2.8% "other" (28). Hispanics of any race made up 3.3% of the population (28). Lexington is 9th among US cities in education level; 39.2% of residents have at least a baccalaureate degree (29). The median annual household income was $39,813 (29), and approximately 13% of the population lived below the federal poverty level (28). Located in Fayette County, the city and county operate as a merged city-county government with a single school district.

### The VERB Summer Scorecard intervention

**Table:** 

**Box. VERB Summer Scorecard Marketing Plan Components**	
**Site**	Lexington, Kentucky
**Target Audience**	Tweens
**Health Focus**	Obesity prevention
**Product Strategy**	Program activities are designed to offer a bundle of benefits: opportunities to have fun, spend time being active with friends, explore new and adventurous activities, and master new skills. An augmented product, the Scorecard, is used to encourage tweens to monitor and try new types of activities.
**Pricing Strategy**	Project activities are designed to overcome tweens' fear of embarrassment in front of their peers and their parents' fears about children's safety, and to provide free or discounted admission to activities.
**Placement Strategy**	A variety of action outlets are offered around the community. These opportunities must comply with the marketing plan and make activity more convenient, safe, and affordable.
**Promotional Strategy**	Free and paid media are used to promote the program. Messages comply with other aspects of the marketing matrix (eg, they do not refer to health benefits associated with physical activity.)

**Figure 1:**
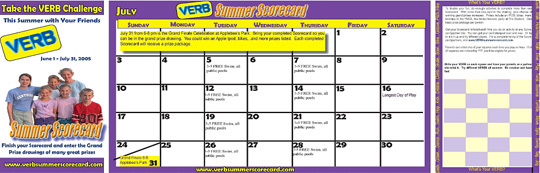
The VERB Summer Scorecard for 2005.

A description of VSS has been published previously (25,26) (see Box for summary). As part of VSS, tweens received a "scorecard" that introduced them to various new activities (ie, free swimming at community pools, strength-training classes, 2-for-1 skating or bowling, and other action-oriented games and events) at community "action outlets" hosted by businesses. The scorecard was wallet-sized and designed to be carried by youth to enable them to track their PA (Figure 1). When youth had been active for at least 60 minutes at an action outlet (or at home), an adult stamped or signed 1 of the scorecard's 24 squares. When all squares were filled, the card could be redeemed for prizes donated by local merchants or other sponsoring organizations, each of which had a PA theme (eg, Frisbee, beach towel, water bottle). Tweens also became eligible for a future "grand prize" — bicycles, martial arts lessons, YMCA memberships, running shoes, and other items that are attractive to tweens. The VSS promotional strategy included media advertisements and appeals to parents and community partners to influence their commitment to providing youth-oriented action outlets.

In the spring of 2004, we recruited multiple organizations in Lexington, including the local parks and recreation department, YMCA, Children's Museum, Boy Scouts, Girl Scouts, faith-based groups, libraries, martial arts studio, and roller and ice skating rinks. Action outlets increased in number during this study, although we did not collect further descriptive data about these venues. We recruited tweens through these organizations and through public schools, camps, and churches. We also contacted parents through parent-teacher association newsletters, workplaces, and churches. The only inclusion criterion for participation was being aged 9 to 13 years. We sent promotional materials to all grade-level–appropriate physical education teachers before disseminating scorecards. We met with principals and asked them to show a 5-minute video via their school-wide system. Announcements were sent to parents via e-mail. All public school students in Lexington who met study age requirements and who attended school were potentially introduced to VSS.

### Instrumentation and assessment of physical activity participation

PA data were collected on 3 occasions. In 2004, 12 public middle schools existed in Lexington, totaling 7,885 students in grades 6 through 8. During autumn 2004, the middle-school version of the Youth Risk Behavior Survey (YRBS-MS) was administered at 8 schools that agreed to be surveyed (30). We modified the YRBS-MS by adding participation items pertinent to VSS: 1) “Have you heard of the VERB Summer Scorecard?”, 2) “Did you get a VERB Summer Scorecard?”, 3) “Did you complete a VERB Summer Scorecard?”, and 4) “During the past 7 days, on how many days were you physically active for a total of at least 60 minutes per day? (Add up all the time you spend in any kind of physical activity that increases your heart rate and makes you breathe hard some of the time.)” This initiative produced the first set of data.

Data collection occurred for the second time in May 2006. A 39-item survey developed by the team of researchers was administered at 9 public and 3 private middle schools. The survey addressed participation in PA; self-efficacy; barriers to and social and parental influences on participation in PA; demographic information; and outcome expectations of and participants' exposure to VERB (18), VERB Yellowball (31), and VSS (32). The YRBS-MS was the source for these PA-related questions.

In 2007, the same schools that had been part of the 2004 YRBS-MS data set were invited to repeat their participation. Six schools agreed, and results from the survey made up the third set of data. The same survey items relevant to PA and VSS used in 2004 were used in 2007.

YRBS-MS items have been scrutinized by measurement experts, some of whom judged the test-retest reliability coefficients (0.55 to 0.68) for PA and sedentary behavior items to be adequate (33) and to have similar prevalence estimates during 2 administrations (34). Other experts found the test-retest intra-class correlation coefficients for moderate PA and VPA items to be 0.51 and 0.46, respectively, among 122 middle school students they examined (35). They also found the sensitivity of the VPA items to be high but to have lower specificity. They concluded that YRBS-MS items may underestimate moderate PA and overestimate VPA among youth (35).

The YRBS-MS was a planned part of the VSS evaluation because few other avenues could reach VSS participants and, more importantly, nonparticipants. The 2004 YRBS-MS administered statewide contained 86 items plus the aforementioned VSS questions. The 2004 VSS evaluation coincided with the Kentucky Department of Education (KDE) survey of middle schools. Coordination of survey collection with the KDE led to using the entire YRBS-MS survey in 2004 and enabled comparison of local and state results inasmuch as few states conduct the YRBS-MS. Conducting the YRBS-MS in 2007 enabled the use of fewer items as well as ones more pertinent to PA and VSS.

### Data analysis

We performed data cleaning and other data management tasks using S-plus version 7.0 for Windows enterprise developer (Tibco Software Inc, Somerville, Massachusetts). Subsequently, we imported data to SAS version 9.1.3 (SAS Institute, Inc, Cary, North Carolina) for analysis. For each year, we performed *Χ* ^2^ tests and computed summary statistics for age, sex, and grade. Chi-square tests and cumulative logit models (Appendix) were used to analyze PA trends in 3 groups: VSS participants, VSS refusers, and VSS reference group students. *Participants* were students who received a scorecard and completed at least some of it. *Refusers* were students who received a scorecard but completed none of it. *Reference group students* were students who reported never having heard of VSS. These models provide odds ratios for activity levels of participants and refusers relative to the reference group students. Missing data were excluded from the analysis, and data for 6,593 students were used in the logit regression analysis. Models did not adjust for socioeconomic status or race/ethnicity because these data were not collected. The Social and Behavioral Science Division institutional review board at the University of South Florida approved the study protocol.

## Results

### Sample

There were 3,428 students surveyed in 2004, 1,976 in 2006, and 2,051 in 2007. The mean age of students was as follows: 2004, 12.2 years (standard deviation [SD], 0.79 y); 2006, 12.3 years (SD, 0.68 y); and 2007, 12.3 years (SD, 0.70 y). Distribution by age and grade differed across years, but sex did not (Table 1).

### Overall frequency of participation in vigorous physical activity

**Figure 2:**
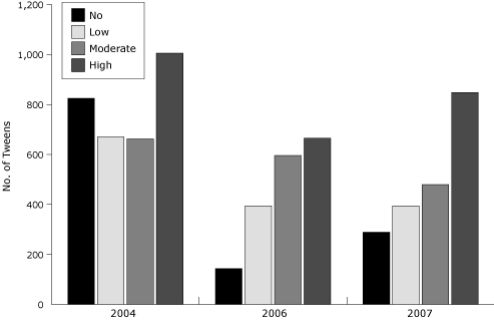
Tweens' participation in vigorous physical activity, by year, Lexington, Kentucky, 2004, 2006, and 2007 (no = 0-1 d/wk, low = 2-3 d/wk, moderate = 4-5 d/wk, high = 6-7 d/wk).

The percentage of students who reported high participation in VPA increased from 31.8% in 2004 to 42.2% in 2007 (*Χ^2^* = 57.7, *P* < .001) (Figure 2). The percentage of students who reported no VPA decreased from 26.1% in 2004 to 14.4% in 2007 (*Χ^2^* = 99.9, *P* < .001).

**Table:** 

**Tweens’ Physical Activity Level**	**No. of Tweens**		

	**2004**	**2006**	**2007**
No (0-1 d/wk)	825	143	289
Low (2-3 d/wk)	670	394	394
Moderate (4-5 d/wk)	662	596	480
High (6-7 d/wk)	1,005	665	848
Total	3,162	1,798	2,011

### Frequency of vigorous physical activity by year and group

In 2004, approximately 24% of VSS participants reported participating in vigorous physical activity at most 1 day per week (Table 2). In 2007, the proportion decreased to approximately 10% (*P* < .001). The proportion of students in the low VPA group (2-3 d/wk) decreased 2.7 percentage points between 2004 and 2007. Although the proportion of students in the moderate VPA category (4-5 d/wk) increased from 2004 to 2006 (20% to 36%) before decreasing to 23% in 2007, the difference between 2004 and 2007 was not significant. From 2006 to 2007, students reporting high VPA (6-7 d/wk) increased by 12.2 percentage points (*Χ^2^* = 13.94, *P* = .002).

The increasing number of VSS participants each year indicates that VSS grew in popularity among tweens during the course of the intervention (Table 2). Furthermore, the increase in the combined proportions of VSS participants reporting moderate and high VPA (16.8 percentage points) was more than twice that for either non-VSS participants or tweens who had never heard of VSS. Finally, compared with tweens who had never heard of VSS, VSS participants were more likely to be active across all levels of VPA than their counterparts.

### Intervention (group) effect

In 2004, there was no difference between VSS participants and tweens in the reference group in terms of frequency of participation in VPA (Table 3). Students in the 2006 reference group were more likely to engage in low to moderate VPA than students in the 2004 reference group. In 2006, odds of engaging in VPA increased for VSS participants overall compared with tweens in the reference group; however, only the odds of engaging in 2 or more days of VPA per week (odds ratio = 1.69), compared with 0 to 1 day per week, was significant. The model also suggests that VSS refusers were less likely to engage in VPA than tweens in the reference group, but this effect did not reach significance. In 2007, VSS participants were 1.6 times as likely as tweens in the reference group to be vigorously active 2 or more days per week, 1.4 times as likely to be vigorously active 4 or more days per week, and 1.4 times as likely to be vigorously active 6 or more days per week.

## Discussion

This study measured the extent to which VPA frequency among youth changed over time and to what extent any observed change could be attributed to the efficacy of VSS. The proportion of tweens who reported *little or no* or *low* VPA decreased from 2004 to 2007, as the proportion of youth who reported *moderate* or *high* VPA increased, regardless of their participation status in VSS. This evidence of a secular trend toward more frequent participation in VPA among youth in Lexington is strengthened by our finding of an increase in youth who reported moderate or high VPA from 2004 to 2007. Furthermore, VSS refusers and tweens from the reference group indicated modest and comparable increases in VPA frequency between 2004 and 2007, despite having either no interest in or awareness of VSS. However, our findings appear to refute the plausibility that observed increases in VPA frequency were a function attributable only to secular trend. The program became increasingly popular over the years, which may indicate that VSS changed youth perceptions of normative behavior in the community.

Our findings suggest that the intervention may have accounted for the observed changes in VPA over the presence of secular trend alone or any other influence unknown to or beyond the control of the investigators. Furthermore, the findings demonstrate how sustained interventions may result in favorable outcomes even if early success with participation is unremarkable and measuring change is methodologically difficult.

The core elements of the intervention may have been crucial to its multiple-year implementation success. Local partnerships helped sustain community interest by providing opportunities for children to be active and broaden intervention awareness, as others have reported (19). Nevertheless, ongoing efforts must develop and expand leadership and strengthen and advance community development to keep the program vibrant (36). The literature indicates that few community-based interventions promote youth PA; therefore, this study and future studies like it have merit (37). Community-based interventions that increase youth PA may be useful in meeting national health objectives of reducing the proportion of children and adolescents who are overweight or obese (38).

This study has limitations. The data represent findings from a single community that may not be representative of others or their youth. Also, there was no comparison community, and local constraints prevented researchers from following the same students throughout data collection years. Without matching, the data were cross-sectional, which made assigning causal inferences inappropriate and eliminated some potentially more robust analytic approaches from consideration. Moreover, only self-report measures were used, so verifying or corroborating accuracy was not possible. Even if respondents were accurate and truthful, reliance on memory introduces the potential of recall bias. Furthermore, we did not track race, ethnicity, or socioeconomic status.

Schools provide a captive audience for obtaining data from tweens. In our experience, however, principals were wary of committing classroom time to surveys unless cash incentives were provided or the resulting data were useful to them in identifying health or safety issues or enabling future grant funding. Coordinating evaluations with planned data collection (ie, the biennial YRBS-MS) allowed researchers, schools, and state agencies to benefit jointly and reduce schools' survey burdens.

These limitations notwithstanding, VSS enables communities to increase youth PA and garner corresponding health benefits. Although the program has spread to other communities, the Lexington coalition chose not to pursue it after 2009, opting instead to concentrate efforts on policy changes to effect change in youth obesity and overweight. Other evaluation initiatives endure in communities where VSS has spread (39). Although incentives were used, a previous VSS evaluation failed to associate incentives with tweens' participation; the act of self-monitoring via the scorecard was the strongest predictor of future intentions to participate in VSS activities (40).

Community-based programs can support and augment school-based prevention programs. School health experts should endeavor to broaden the effect of PA messages and provide continuity with the VSS strategy that PA is fun and not merely an approach to prevent obesity or its risk factors.

## Figures and Tables

**Table 1:** Demographic Composition of Lexington, Kentucky, Students, by Year^a^

Characteristic	Year, n (%)			*X* ^2^	*P* Value

	2004	2006	2007		
**Age, y**					
10	16 (0.5)	3 (0.2)	7 (0.3)	143.1	<.001
11	766 (22.3)	239 (12.1)	261 (12.7)		
12	1,194 (34.8)	860 (43.5)	849 (41.4)		
13	1,452 (42.4)	874 (44.1)	934 (45.5)		
**Grade**					
6	1,225 (36.0)	966 (48.6)	1,006 (49.3)	332.6	<.001
7	1,300 (38.2)	841 (42.3)	796 (39.0)		
8	881 (25.8)	181 (9.1)	240 (11.8)		
**Sex**					
Male	1,719 (50.3)	969 (48.9)	1,024 (50.0)	1.08	.58
Female	1,697 (49.7)	1,014 (51.1)	1,026 (50.0)		

a Values for n may not sum to the total number of students surveyed each year because of missing demographic (<2%) and vigorous physical activity (8%) data.

**Table 2:** Frequency of Vigorous Physical Activity, by VERB Summer Scorecard (VSS) Participation Group and Year^a^

Group	Year	Frequency of Vigorous Physical Activity,^b^ N (%)				n	*X* ^2^	*P* Value

		Little/No	Low	Moderate	High			
Participants	2004	63 (23.7)	52 (19.6)	52 (19.6)	99 (37.2)	266	93.3	<.001
	2006	17 (4.1)	90 (21.8)	149 (36.1)	157 (38.0)	413		
	2007	51 (9.6)	90 (16.9)	125 (23.4)	268 (50.2)	534		
Refusers	2004	176 (22.5)	163 (20.8)	167 (21.4)	276 (35.3)	782	75.5	<.001
	2006	50 (7.8)	147 (22.8)	218 (33.8)	230 (35.7)	645		
	2007	158 (14.8)	222 (20.8)	268 (25.1)	420 (39.3)	1,068		
Never Heard of VSS^c^	2004	506 (26.7)	410 (21.6)	406 (21.4)	574 (30.3)	1,896	92.8	<.001
	2006	59 (9.6)	133 (21.7)	189 (30.8)	233 (37.9)	614		
	2007	75 (20.1)	75 (20.1)	80 (21.4)	144 (38.5)	374		

a Values for n may not sum to the total number of students surveyed each year because of missing demographic (<2%) and vigorous physical activity (8%) data.

b Little/No = 0-1 d/wk, Low = 2-3 d/wk, Moderate = 4-5 d/wk, High = 6-7 d/wk.

c Reference group.

**Table 3:** Odds of Vigorous Physical Activity, by Group and Year, VERB Summer Scorecard Evaluation, Lexington, Kentucky

**Parameter**	FN^a^	OR	Estimate	SE	*Χ* ^2^	*P* Value
Intercept	1	NA	1.85	0.05	1557.21	<.001
	2	NA	0.59	0.03	373.11	<.001
	3	NA	−0.49	0.03	276.38	<.001
**Year**						
2006	1	2.16	0.77	0.08	99.48	<.001
	2	1.35	0.30	0.04	46.2	<.001
	3	0.97	−0.03	0.04	0.53	.46
2007	1	0.94	−0.06	0.06	0.86	.35
	2	1.09	0.09	0.04	3.96	.046
	3	1.21	0.19	0.04	21.82	<.001
**Group/year**						
Participants 2006	1	1.69	0.53	0.18	8.7	.003
	2	1.18	0.16	0.09	3.68	.05
	3	1.03	0.03	0.08	0.18	.67
Refusers 2006	1	0.86	−0.15	0.14	1.15	.28
	2	0.94	−0.07	0.07	0.79	.37
	3	0.93	−0.07	0.07	0.94	.33
Participants 2007	1	1.57	0.45	0.11	16.75	<.001
	2	1.42	0.35	0.08	20.72	<.001
	3	1.36	0.31	0.07	18.61	<.001
Refusers 2007	1	0.96	−0.04	0.09	0.25	.62
	2	0.92	−0.08	0.06	1.54	.21
	3	0.87	−0.14	0.06	4.79	.03
Participants 2004	1	1.03	0.03	0.10	0.09	.76
	2	1.07	0.07	0.09	0.64	.42
	3	1.14	0.13	0.09	2.15	.14
Refusers 2004	1	1.10	0.10	0.08	1.62	.20
	2	1.07	0.07	0.07	1	.32
	3	1.05	0.05	0.07	0.51	.47

Abbreviations: FN, function number; OR, odds ratio; SE, standard error; NA, not applicable; VPA, vigorous physical activity.

a The first function number shows the odds of having at least low frequency VPA (2-3 d/wk) by group and year. The second function number shows the odds of having at least moderate VPA (4-5 d/wk) by group and year. The third function number shows the odds of having high VPA (6-7 d/wk) by group and year.

## Appendix. Cumulative Logit Models Used to Analyze Physical Activity Levels of Tweens

The response variable used 4 categories in which tweens (youth aged 9-13 y) had been grouped (according to the number of days per week they reported vigorous physical activity [VPA]). Thus, in the notation below, category 0 represents *little or no* VPA (0-1 d/wk), category 1 is *low* VPA (2-3 d/wk), category 2 is *moderate* VPA (4-5 d/wk), and category 3 is *high* VPA (6-7 d/wk). Using *P*_0_, *P*_1,_ *P*_2_, and *P*_3_ to represent the population proportion of students in each category, the analysis used the following 3 logits:

The first logit (or response function) describes the log odds of having *at least* *low* frequency of VPA as opposed to *little or no* VPA. The second logit describes the log odds of having at least *moderate* VPA as opposed to *low* VPA or *little or no* VPA. The third logit represents the odds of having *high* VPA compared with all other categories of VPA. The 3 logits were modeled simultaneously with respect to the intervention effects. For example, the following model considers the effect of the VSS intervention on the distribution of frequencies of VPA among students in a given year. The variable group classified students according to whether they were in the reference group, the refusers group, or the participants group. Because the assessment took place in 3 waves (years), the "group" effects are more appropriately evaluated for the cohort of students in each year. Thus, "year" is a main effect in the model and "group" is nested within "year." Information at the class level was not available. In the set of formulas shown below, the first calculates the odds of engaging in VPA on 2 or more days per week; the second calculates the odds of engaging in VPA on 4 or more days per week; and the third calculates the odds of engaging in VPA on 6 or more days per week:
